# An Optimized Multi‐Stage Drainage Cannula Design for Venoarterial Extracorporeal Membrane Oxygenation

**DOI:** 10.1111/aor.70203

**Published:** 2026-07-19

**Authors:** Avishka Wickramarachchi, Mehrdad Khamooshi, Aidan Burrell, Shaun D. Gregory

**Affiliations:** ^1^ Advanced Cardiorespiratory Engineering Laboratory, Faculty of Engineering Queensland University of Technology Brisbane Australia; ^2^ Centre for Biomedical Technologies, School of Mechanical, Medical and Process Engineering, Faculty of Engineering Queensland University of Technology Brisbane Australia; ^3^ Cardio‐Respiratory Engineering and Technology Laboratory, Department of Mechanical and Aerospace Engineering, Faculty of Engineering Monash University Clayton Australia; ^4^ Department of Intensive Care Alfred Hospital Melbourne Victoria Australia; ^5^ Australian and New Zealand Intensive Care Research Centre Monash University Melbourne Victoria Australia

**Keywords:** CFD, differential oxygenation, flow dynamics, optimization, thrombosis, VA ECMO

## Abstract

**Background:**

Venoarterial extracorporeal membrane oxygenation (VA ECMO) is a life‐saving therapy that provides cardiorespiratory support for cardiogenic shock patients. But, due to altered flow dynamics induced by VA ECMO drainage cannulas, complications such as thrombosis and hemolysis are common. Therefore, this study aimed to develop an optimized drainage cannula design with reduced complication risk using computational fluid dynamics (CFD).

**Methods:**

The multi‐objective optimization process entailed generating a response surface based on a design of experiments with *n* = 540 unique cannula designs. Minimizing pressure drop, maintaining > 100 mm/s tip velocity, and minimization of average wall shear stress were assigned as objective functions. The optimized design was then tested in three patient‐specific geometries using CFD and compared against a clinically available model (Maquet).

**Results:**

The final design consisted of three rows of five side holes, with all side holes titled at an angle of 31.5°. When compared to the Maquet model, the optimized cannula generated lower stagnant blood volumes, increased cannula tip velocities (> 300 mm/s), and increased drainage from the upper body.

**Conclusion:**

The optimized cannula developed in this study demonstrated favorable flow dynamics that may increase flow and reduce the risk of life‐threatening complications including thrombosis and hemolysis when translated to a clinical setting, thereby improving VA ECMO patient outcomes.

## Graphical abstract

This study utilized an extensive optimization involving *n* = 540 design of experiments to produce a superior drainage cannula design. Compared to the clinically used Maquet model, our optimized design demonstrated reduced blood stagnation volumes, increased cannula tip velocities, and higher upper body drainage, potentially minimizing risk of thrombosis and differential hypoxia.

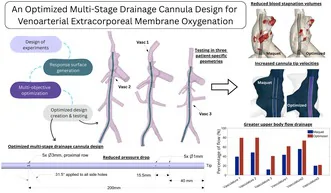

## 1 Introduction

Venoarterial extracorporeal membrane oxygenation (VA ECMO) utilizes an externally placed pump and oxygenator to provide circulatory and respiratory support for refractory cardiogenic shock patients. Venous return from the patient is transferred to the pump using a peripherally inserted drainage cannula. Although many cannulation strategies exist, the drainage cannula is commonly advanced from the femoral vein to the right atrium (RA) or superior vena cava (SVC) [1]. Design of a drainage cannula involves an end hole at the tip and side holes throughout the cannula to enable drainage from various points in the venous system. The importance of drainage cannula side holes has been extensively evaluated in the literature, with multiple studies demonstrating that a bulk of venous flow is drained by the side holes closest to the pump (proximal side holes) [2]. This predisposes drainage cannulas to many complications that ultimately contribute to patient mortality.

A common drainage cannula associated complication is thrombosis; the formation of blood clots [3, 4]. These clots can form around or within the cannula and are at high risk of embolization, particularly during or after decannulation. This can lead to blockage of the distal vessels, thereby causing limb ischemia, travel through the circuit leading to blockage of the oxygenator, risking reduced circuit flows, or, in some rare cases, movement through the tricuspid valve, leading to occlusion of the pulmonary system vessels, causing respiratory distress [4, 5]. As blood is primarily drained by the proximal side holes, blood flow in and around the cannula tip is at risk of stagnating, thereby inciting the coagulation cascade [6, 7]. In fact, cannulas with a higher number of side hole rows have been shown to generate increased volumes of stagnant blood near the cannula tip [7]. Additionally, reliance on the proximal side holes for drainage increases the risk of infra‐renal inferior vena cava (IVC) collapse. Vessel collapse can cause endothelial lining damage and damage to red blood cells (hemolysis) [8]. It is hypothesized that increasing flow through the cannula tip may reduce vessel collapse risk since venous return from the upper body is available for additional drainage [1].

Reduced cannula tip flows also reduce the amount of flow extracted from the upper body via SVC venous return, and this phenomenon is exacerbated in cannulas with proximal side holes further away from the RA [7]. Consequently, upper body venous return is recirculated back through the body via the tricuspid valve, instead of being drained to the VA ECMO circuit. This can have severe consequences, such as worsening of a complication called differential oxygenation and hypoxemia. This is when patients with respiratory dysfunction have an upper body that is poorly oxygenated, but a lower body that is adequately oxygenated. In such patients, flow supplied to the upper body organs, such as the brain, is trapped in a circulation that is not being oxygenated by VA ECMO support, leading to adverse neurological outcomes [9]. Although this complication is primarily associated with arterial cannula flow dynamics due to the interaction between oxygenated VA ECMO flow and deoxygenated native ejection, unfavorable drainage cannula flow characteristics, such as insufficient superior vena cava drainage, can also exacerbate the complication. Jugular insertion of the drainage cannula may alleviate the risk of differential oxygenation [10], but femoral cannulation is still the preferred and most commonly used method. Therefore, a femoral venous cannula that can drain adequate amounts of venous return from the upper body is desirable.

An often‐overlooked factor in cannula related studies is that the pump in a VA ECMO circuit is the main source of platelet activation and hemolysis [11]. Therefore, reducing the shear rates within the pump may minimize the risk of blood trauma. This can be done via less resistive cannulas, thereby enabling lower pump operating speeds [11, 12]. Previous research has shown that the positioning and size of the drainage cannula's side holes impact the cannula's flow capabilities [6]. Therefore, appropriate manipulation of side hole design can be exploited to reduce the overall circuit resistance, thus minimizing the risk of pump‐associated trauma.

Evidently, the design of a drainage cannula has key implications towards risk of life‐threatening complications for patients receiving VA ECMO. To this end, there has been growing interest in the improvement of drainage cannula design, with the number of side holes, side hole size and angle, and inner cannula wall shape being investigated and also optimized [13, 14, 15]. However, many of these studies only focus on improving single‐stage cannulas, where the side holes are clustered near the tip. Such cannulas are less frequently used clinically, in favor of multi‐stage cannulas, where the side holes are distributed over larger distances throughout the cannula. Yet, there are no studies that have optimized multi‐stage drainage cannula design. Therefore, the aim of our study was to utilize a computational fluid dynamics (CFD) informed parametric design optimization process to alter a multi‐stage drainage cannula design towards reduced thrombosis and differential oxygenation risk while improving its hydraulic characteristics to reduce overall circuit resistance.

## 2 Methods

### 2.1 Geometry, Meshing, and Boundary Conditions

#### 2.1.1 Optimization Geometry

The optimization workflow was conducted in an idealized geometry of the RA, SVC, and IVC, similar to those employed in other studies [15, 16]. A 21 Fr drainage cannula was concentrically placed, with its axis parallel to the IVC‐SVC cylinder. The specific size and design of the cannula reflected the 21 Fr HLS Maquet (Getinge) of 55 cm length, a commonly used drainage cannula, particularly in our institution [17]. The cannula length modeled in this study was 400 mm, whereas the distance from the tip to the proximal row was 200 mm. The cannula contained five rows of side holes. Each row consisted of four 2.5 mm diameter side holes. This idealized geometrical set up (Figure 1A) was chosen to simplify the geometry of the cannula and surrounding vasculature, thereby excluding tendencies to bias optimization towards one patient‐specific vasculature and other complexities such as cannula curvature. Details regarding meshing and mesh‐independence data for this geometry can be found in Supporting Information.

**FIGURE 1:**
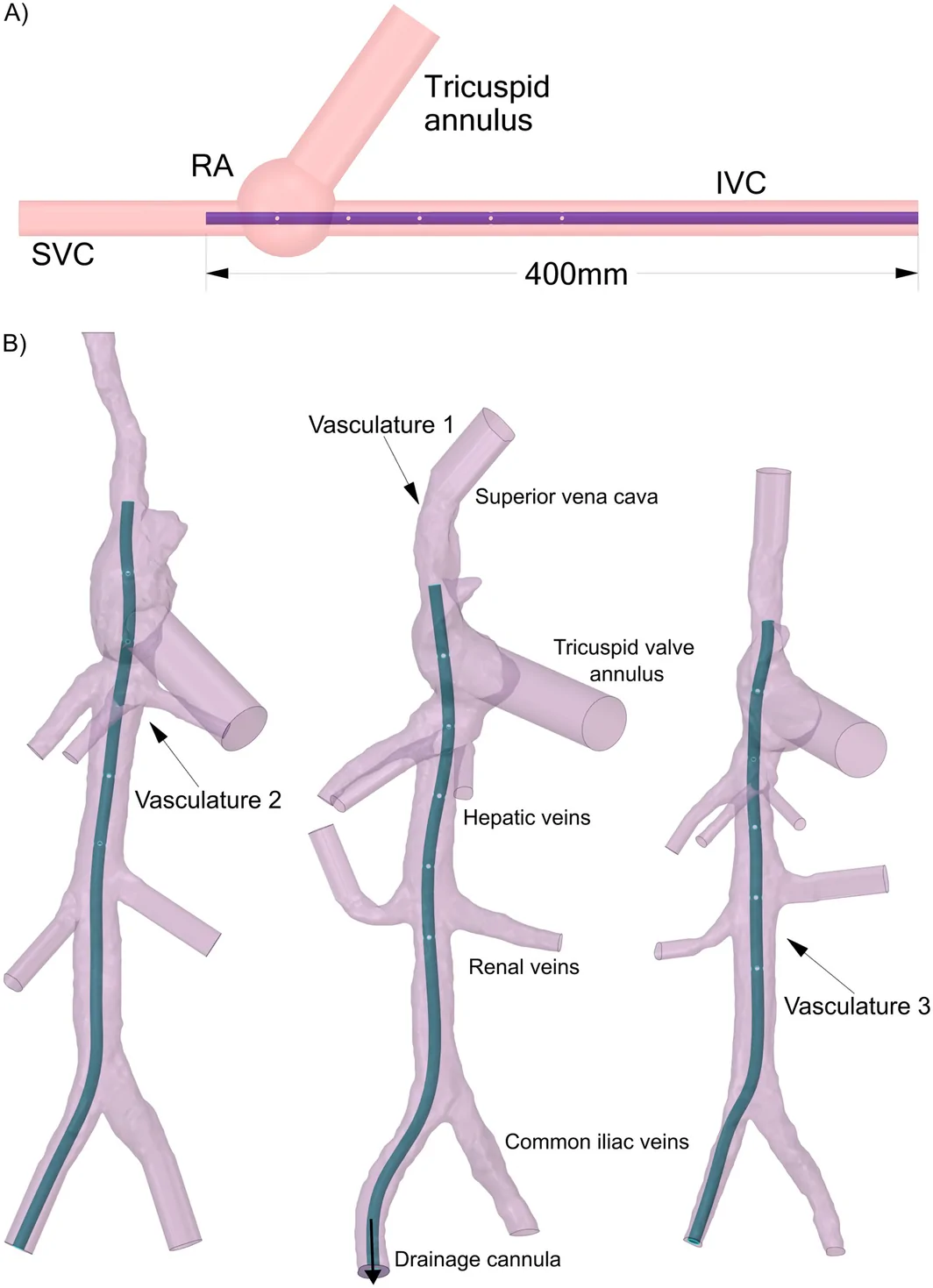
Idealized geometry used for the optimization strategy (A) and the three patient‐specific geometries used in the evaluation stage with a 21 Fr Maquet HLS cannula modeled within each vasculature (B).

IVC and SVC inlet flow rates were assigned as 3.85 and 1.15 L/min, respectively. This combines the flow rates imposed by each major vein when utilized in a patient‐specific geometry (as done previously [6, 7, 18], and taken from Heldt et al. [19]). The cannula was then assigned a 4 L/min drainage flow rate, reflecting standard VA ECMO support levels [20], whereas the tricuspid annulus was assigned a zero‐pressure condition [6, 7, 18]. Blood was modeled as non‐Newtonian using the Carreau model (Equation 1), and all optimization simulations were performed using the *k* − *ω* SST turbulence model in a steady state setting, with convergence criteria set to 10^−4^ for all residuals.

(1) $$\eta =\eta_{\infty }+\left(\eta_{0}-\eta_{\infty }\right){\left(1+\gamma^{2}\lambda^{2}\right)}^{\frac{n-1}{2}}$$

where *η* is the local viscosity, *η*_∞_ = 0.00345 kg/(m.s), *η*_0_ = 0.056 kg/(m.s), *γ* is the local shear rate, *λ* = 3.313 s, and *n* = 0.3568 [21].

#### 2.1.2 Patient‐Specific Geometries

The developed cannula was then tested in three separate patient specific geometries (Figure 1B), taken from CT scans of VA ECMO patients (with ethics approval from the Alfred Hospital—Project 70/19) to verify the improvements produced by the optimized design. These patients were chosen under strict guidance from clinical experts at our institution to reflect venous vasculatures of varying size to evaluate the effectiveness of the optimized design in different environments. The extraction process was performed using MIMICS 21.0 image segmentation software (Materialize, Leuven, Belgium). A 21 Fr Maquet HLS cannula was chosen as the control design for comparison, and both cannulas were placed along centerlines of each venous geometry with the tip lying in the SVC‐RA junction [1].

Mesh settings and outlet boundary conditions for the patient specific geometries were performed with identical settings to those applied in previous studies [6], resulting in mesh sizes between 2.6 and 3.5 million polyhedral elements. Further meshing details can be found in Supporting Information. The constant inflow rates from each vein were imposed similar to those done in prior studies. In particular, the SVC's mass inflow rate was assigned 23% of the total 5 L/min venous return. However, some studies have reported variation in upper body venous return, which may alter right atrial flow structures and blood stagnation volumes. For instance, some studies suggest SVC inflow rates provide 35% of venous return [22]. Therefore, to account for such variations, two sets of simulations were performed with the specific flow rates outlined in Table 1. The first (SVC‐23) utilized the parameters set by Heldt et al. [19], as used in a baseline cardiovascular lumped parameter model. The other set of simulations utilized a 35% SVC inflow condition (SVC‐35). All simulations controlled total venous return to be 5 L/min.

**TABLE 1:** Inflow boundary conditions assigned to the major veins for SVC‐23 and SVC‐35 scenarios.

Sub‐circulation	SVC‐23		SVC‐35	
	Percentage of CO (%)	Total flow rate (L/min)	Percentage of CO (%)	Total flow rate (L/min)
Upper body	23	1.15	35	1.75
Splanchnic	30	1.5	26	1.3
Renal	22	1.1	18	0.9
Lower body	25	1.25	21	1.05

Abbreviations: CO, cardiac output; SVC, superior vena cava.

The simulations utilizing patient‐specific geometries were run in a unsteady Reynolds‐averaged Navier–Stokes (URANS) setting with the Maquet and optimized cannulas for 10 s at a timestep of 1 ms. Again, the *k* − *ω* SST turbulence model was used, blood was modeled as a non‐Newtonian fluid using the Carreau model, and the convergence criteria were set to 10^−4^ for all residuals during each timestep. All simulations were performed using ANSYS Fluent 24R2 during this study.

#### 2.1.3 Cannula Performance Assessment

Several markers for thrombosis risk were measured to assess hemocompatibility. First, volumes of stagnant blood within the CFD domain were measured as per the following criteria: volumes of blood where velocity magnitudes < 0.01 m/s and shear rates < 2/s, as previously used by our group [6, 7]. These abnormally low shear regions are prone to inciting the coagulation cascade and eventual clot formation.

Another marker indicative of potential thrombosis risk is poor clearance of blood out of the venous geometry. Fast washout of blood through the cannula and tricuspid annulus prevents persistence of old blood within the domain, thereby reducing prolonged blood residence. For this study, a scalar variable, of value one, populated the CFD domain upon simulation initialization. Then, a scalar with value zero was assigned to blood entering the domain via the inlets. Washout was then measured by calculating the volume of blood with a scalar value > 0.5 when compared to the total CFD blood volume, thereby providing an indication of old blood displacement [6].

Blood trauma caused by the cannulas was measured via calculation of hemolysis indices (HI). Specifically, the following power law equation was used:

(2) $$\begin{array}{c} \mathrm{HI}\%=\frac{∆H_{b}}{H_{b}}\times 100=Ct^{a}\tau_{s}^{b} \end{array}$$

where Δ*𝐻*_b_ is the concentration of plasma‐free hemoglobin, *H*_b_ is the concentration of total hemoglobin, *t* is exposure time, *𝜏*_s_ is shear stress, and *C*, *α*, and *𝛽* are constants obtained from Zhang et al. [23].

At last, the amount of SVC flow and, therefore, upper body venous return drained by the cannulas was measured. This was done by seeding massless particles to SVC flow and measuring the percentage of particles that exited via the cannula in relation to total particles seeded [7, 18].

### 2.2 Optimization Settings

The optimization process is highlighted by the flowchart depicted in Figure 2. The design of experiments (DoE) generates different cannula geometries by varying the input parameters between the allowable values. The parameterization, input parameters, and geometrical constraints for the cannula geometry are extensively described in Supporting Information. Each geometry is then simulated under identical flow boundary conditions, saving the key results (output parameters) at the end of the simulation. The output parameters for our study were pressure drop, cannula tip velocity, and average wall shear stress to align with the optimization objectives. Other parameters could have been considered, but would greatly increase computational requirements for this study. To populate the input parameters for the DoE, an optimal space filling algorithm was used which distributed the design parameters equally throughout the design space between the allowable ranges. To ensure sufficient points were simulated to generate an accurate response surface, 540 design points populated the DoE.

**FIGURE 2:**
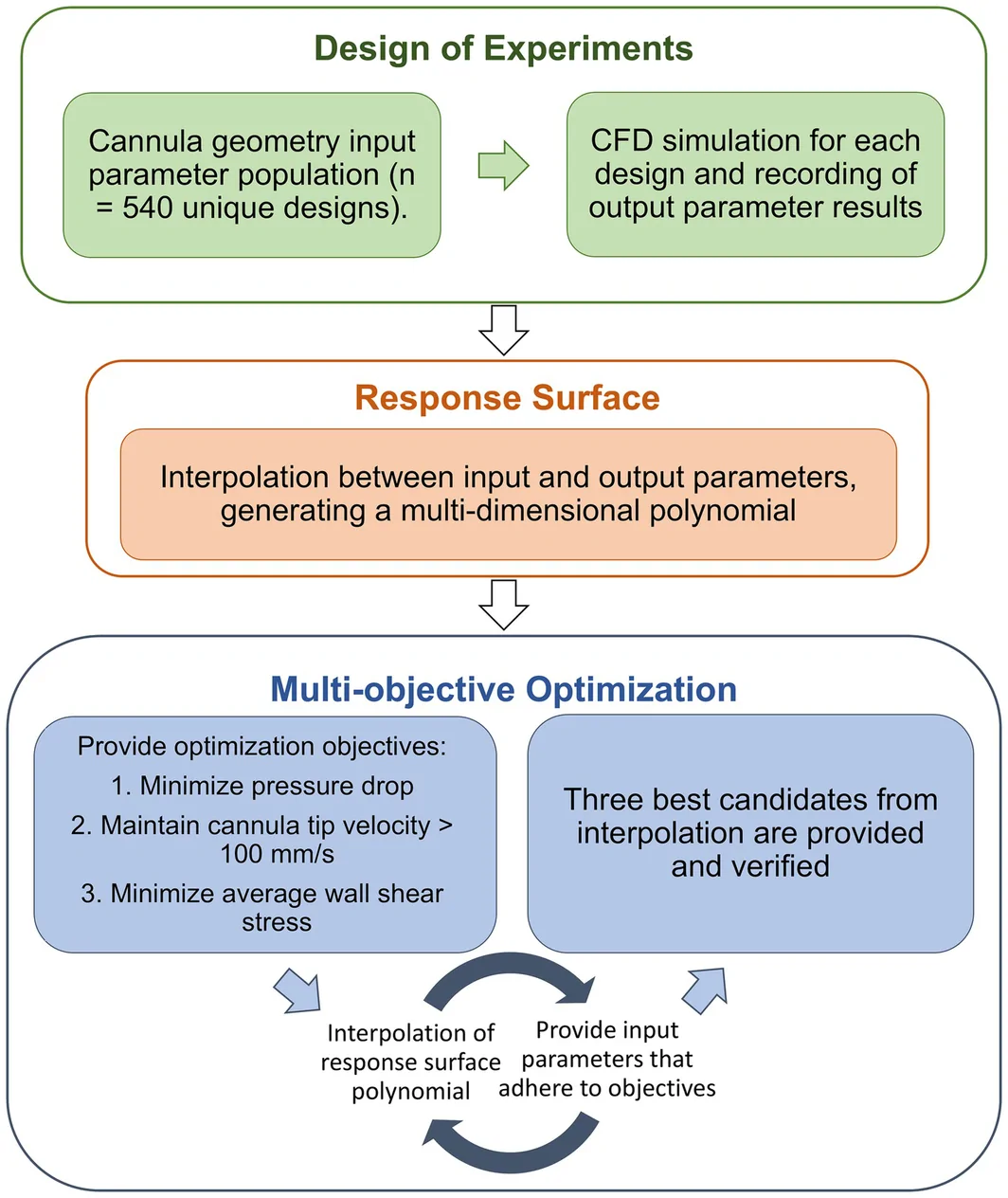
Diagram indicating the optimization process used to generate the final cannula design.

The response surface is a pseudo‐approximation model that is a best‐fit curve through all data points from the DoE. Therefore, it is able to form a relationship between input and output parameters, which can then be used to perform approximations without performing CFD simulations. A Kriging response surface was utilized, thereby generating a multi‐dimensional polynomial that fit through all 540 design points. Fifty additional refinement CFD simulations were run to improve the accuracy of the Kriging model below 5% overall error.

At last, a multi‐objective optimization component employed the generated response surface to produce unique cannula designs based on user input objectives. For our study, we set this to be:

- Minimization of pressure drop (strict priority).
- Increase cannula tip velocity (threshold of > 100 mm/s).
- Minimization of average wall shear stress.

A cannula with a reduced pressure drop is desirable from a clinical perspective. It allows for lower pump speeds to be used to achieve adequate VA ECMO support, which may also reduce pump‐related blood trauma. Therefore, minimization of pressure drop was assigned as an objective with a strict adherence priority and calculated as the pressure difference between the tip and end face of the drainage cannula. The coordinates for each surface center were obtained from the geometry file for each vasculature and all pressures were calculated as the area‐averaged pressure for each surface. Low cannula tip velocities may contribute to increased blood stagnation risk and lower upper body drainage [1, 7]. Therefore, to reduce these undesirable effects, a velocity magnitude threshold of > 100 mm/s was assigned to the cannula tip. This value was chosen due to previous studies by our group highlighting increased blood stagnation volumes with cannulas that had tip velocities < 100 mm/s [7]. At last, minimization of average wall shear stress was chosen as the last objective to reduce the accumulated shear contribution towards thrombosis risk. It was assigned a lower priority to prevent conflict with the pressure drop objective as we expect pump‐related shear stresses to contribute a much larger portion towards blood trauma than shear stress within the cannula [11]. The best input parameters that adhere to the set objectives were then chosen to form the optimized cannula design.

## 3 Results

The multi‐objective optimization workflow produced a final drainage cannula design as depicted in Figure 3. Unlike the Maquet cannula, the optimized design only had three rows of side holes, with each row consisting of five side holes. In the Maquet cannula, all side holes protrude perpendicular to the cannula's length. However, the optimized cannula had side holes that were tilted by 31.5° to this perpendicular axis. The diameters of the side holes in the proximal row were also larger (3 mm) when compared to those in the rows near the tip (1 mm).

**FIGURE 3:**
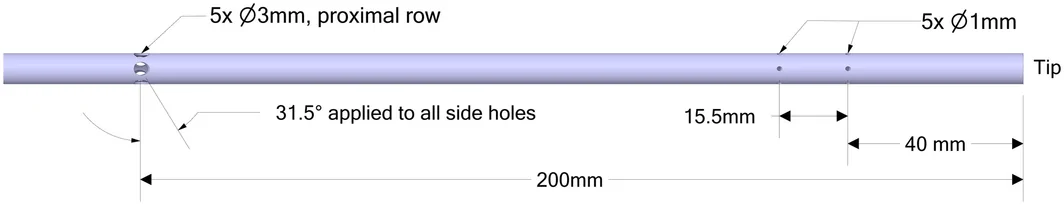
Truncated version of the final candidate design produced by the optimization process.

Several parameters were then measured when testing the optimized cannula in the patient‐specific geometries to confirm the improvements predicted by the response surface approximations.

### 3.1 Pressure‐Drop

The optimized drainage cannula consistently had a lower pressure drop than the Maquet cannula throughout all simulations when draining a set flow rate of 4 L/min (Table 2). It is important to note that the pressure drop varies between the three patient‐specific geometries due to the difference in length of the modeled cannula. Furthermore, these pressure‐drop results do reflect the absolute values that would be observed clinically as the full cannula length is not modeled within the CFD domain. Instead, we are comparing the difference in pressure drop that arises purely due to the variation in cannula design. Overall, the average decrease in pressure drop was 7.66 ± 0.29 mmHg during all simulations between the optimized and Maquet cannula. Negligible differences were observed in each cannula between SVC‐23 and SVC‐35 simulations as the difference in SVC flow did not impact the drainage performance of the cannula itself, provided that total venous return was 5 L/min in both cases.

**TABLE 2:** Pressure drop results for the optimized and Maquet cannulas.

Geometry	Pressure drop (mmHg)			
	SVC‐23		SVC‐35	
	Maquet	Optimized	Maquet	Optimized
Vasculature 1	78.9	71.3	79.2	71.1
Vasculature 2	85.2	77.5	85.2	77.5
Vasculature 3	71.7	64.0	71.1	64.0

### 3.2 Tip Velocities

The optimized cannula generated tip velocities > 300 mm/s throughout all simulations (Figure 4). These tip velocities were up to 6.6 times higher than those observed in the Maquet cannula and have the potential to reduce the risk of flow stagnation at the tip. All tip velocities were higher during the SVC‐35 simulations and can be attributed to increased SVC flow. This was particularly evident in the Maquet cannula results, with an increase of 34 mm/s observed in Vasculature 3.

**FIGURE 4:**
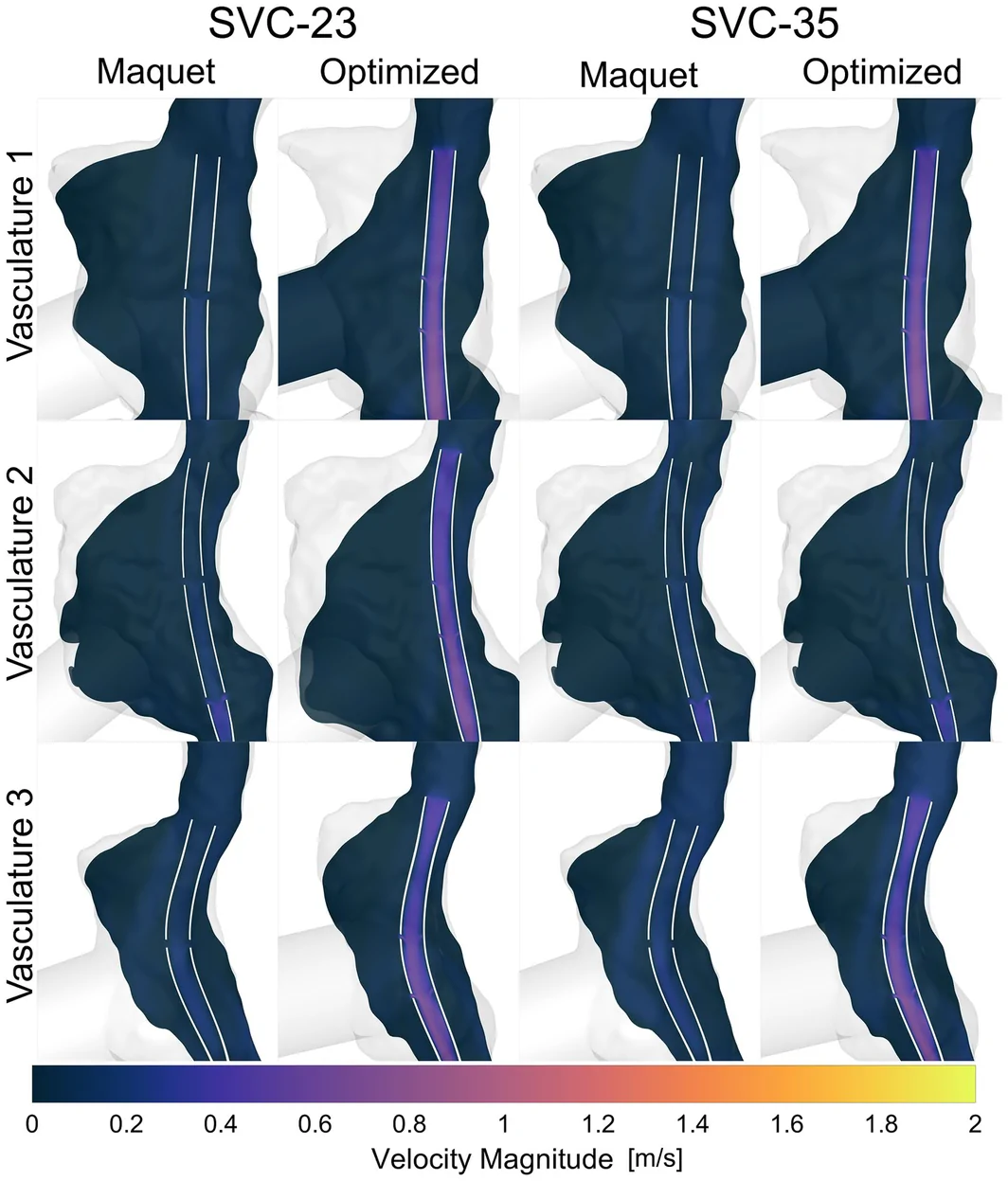
Contours of velocity magnitude through the distal tip end of the Maquet and optimized cannulas.

### 3.3 Blood Stagnation Volume

The optimized cannula generated lower volumes of stagnant blood during most (5/6) simulations, when compared to the Maquet cannula (Figure 5). This can be attributed to increased drainage via the tip and distal side holes in the optimized cannula. Larger volumes of stagnant blood formed primarily in the right atrial appendages during the SVC‐23 simulations. The optimized cannula presented a larger blood stagnation volume than the Maquet cannula in Vasculature 1 when SVC‐35 inflow settings were applied (9.34 vs. 6.69 mL). This may be due to excessive upper body drainage, preventing adequate washout of the atrial appendage. A decrease in blood stagnation volumes was observed with both cannulas in all other simulations with SVC‐35 settings, when compared to SVC‐23 simulations. The less pronounced appendage of Vasculature 3 resulted in similar and lower stagnant volumes for both cannulas during SVC‐23. Overall, neither the Maquet nor optimized design demonstrated blood stagnation within or directly around the cannula.

**FIGURE 5:**
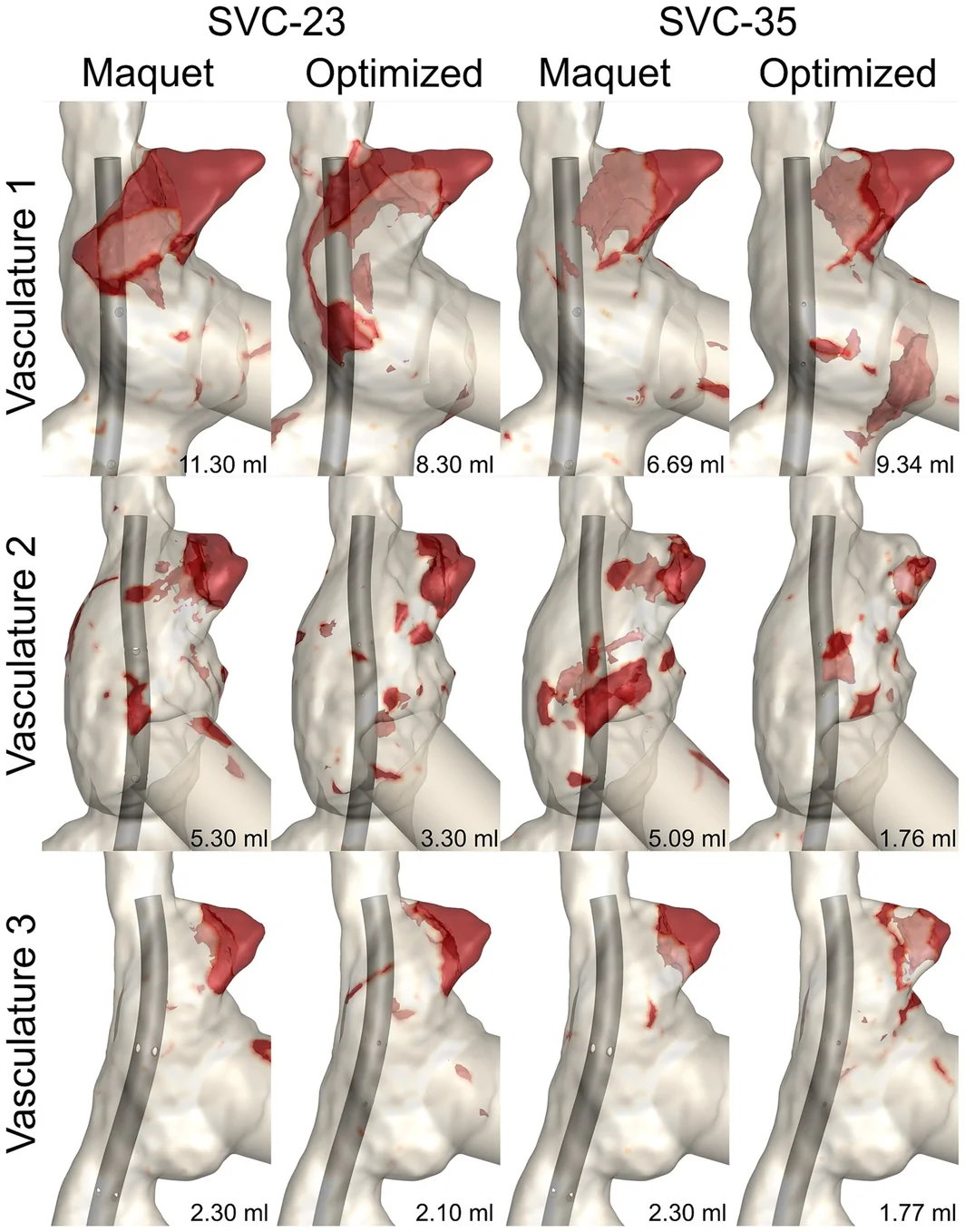
Blood stagnation regions and stagnant volume values during SVC‐23 and SVC‐35 simulations for the Maquet and optimized drainage cannula designs.

### 3.4 Rates of Washout

Washout rates measure how fast residual blood is cleared from the CFD domain by new blood entering via the major veins. Complete washout would return a residual blood volume of 0%. In Vasculature 1, the optimized cannula performed superiorly during SVC‐23 settings but provided no significant improvement after 10 s during the SVC‐35 scenario (Figure 6). The optimized cannula also demonstrated faster washout when placed in Vasculature 2 during SVC‐23 and SVC‐35 scenarios. At last, both cannulas depicted negligible differences in washout when placed in Vasculature 3, regardless of SVC inflow conditions.

**FIGURE 6:**
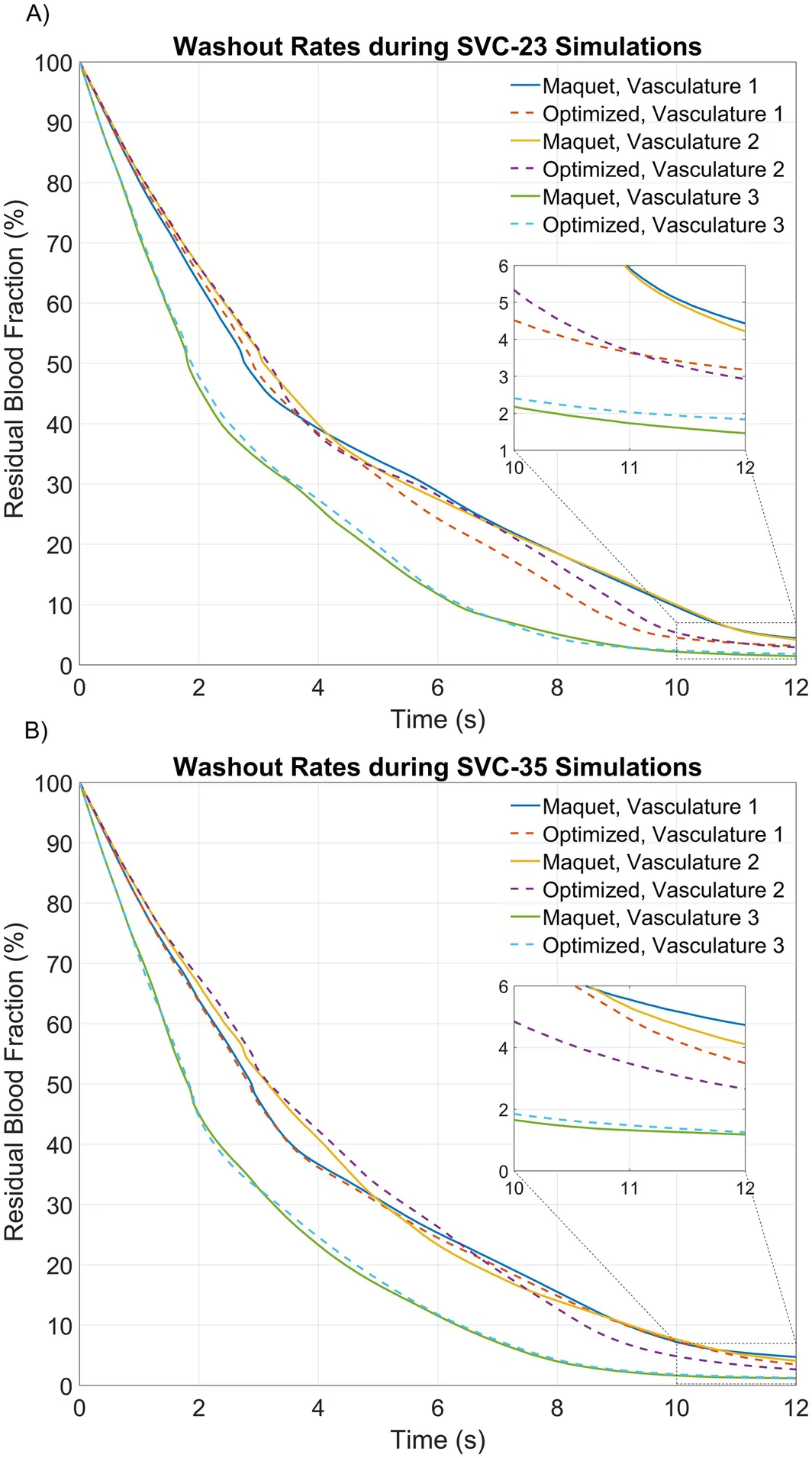
Rates of washout between the Maquet and optimized cannulas in each geometry during the SVC‐23 (A) and SVC‐35 (B) simulations. Here 0% would refer to complete displacement of old residual blood with new blood entering the system.

### 3.5 Hemolysis

Measurement of hemolysis indices produced by both cannulas revealed lower or equal risk of hemolysis with the optimized design than the Maquet cannula (Figure 7). The largest difference occurred when both cannulas were placed in Vasculature 1 under SVC‐23 conditions, with the Maquet cannula producing a hemolysis index of 1.06 × 10^−3^ %, compared to 1.02 × 10^−3^ % with the optimized design. However, these improvements are small, and thus it is difficult to claim any superiority of the optimized design over the Maquet cannula.

**FIGURE 7:**
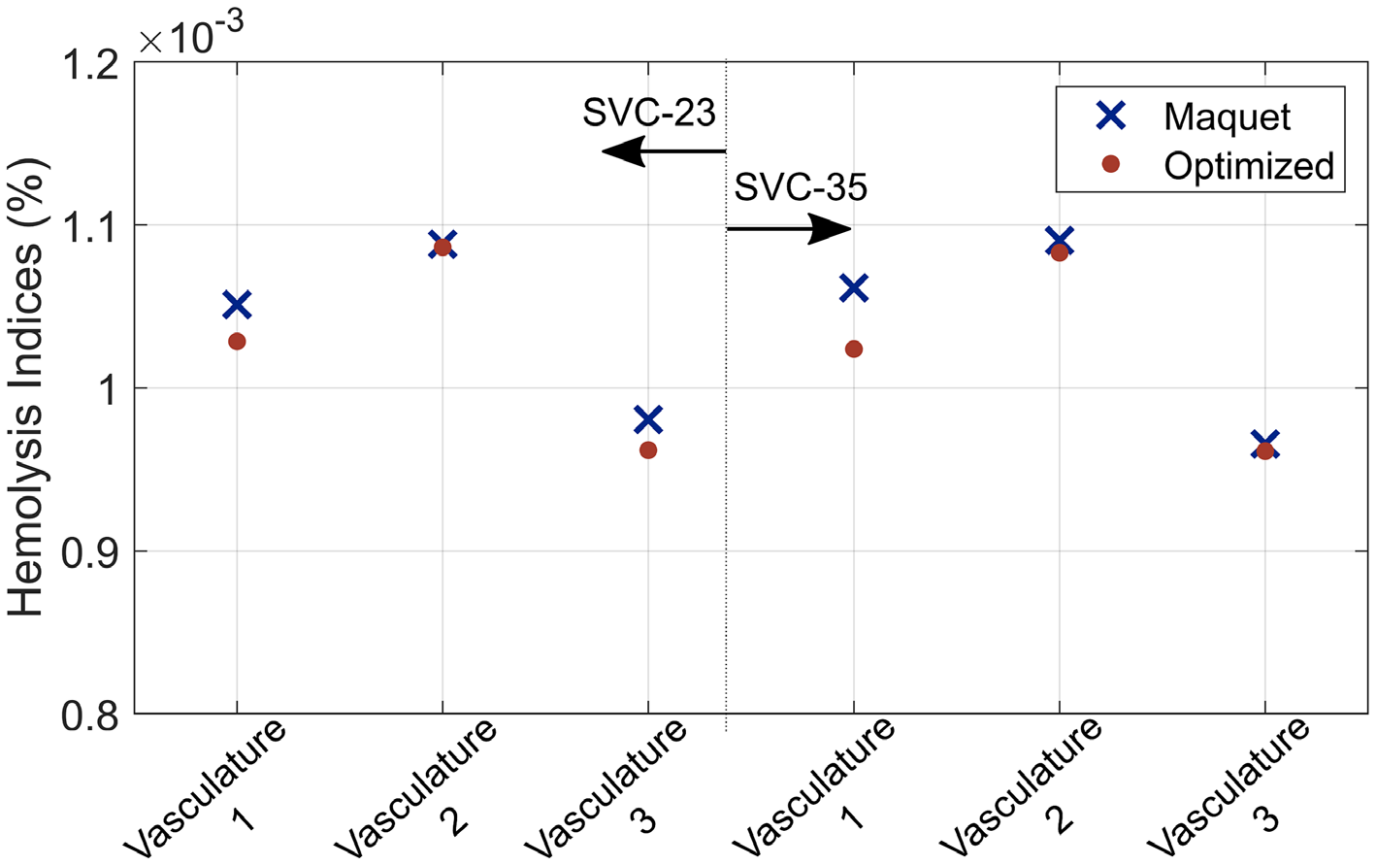
Indices of hemolysis for the Maquet and optimized cannulas.

### 3.6 Upper Body Drainage

The amount of flow extracted from the SVC was recorded to measure efficacy of drainage from the upper body circulation. This is of particular importance as a lack of sufficient upper body drainage may increase the risk of developing differential oxygenation. The optimized cannula drained a greater portion of flow from the upper body than the Maquet cannula during all simulations (Figure 8). The remaining SVC flow passed through the tricuspid annulus, therefore indicating passage through to the pulmonary circulation and beyond. Interestingly, both the Maquet and optimized designs performed poorly when placed in Vasculature 3 during the SVC‐35 scenario (Maquet: 19.9% vs. optimized: 21.6%). This may be attributed to the combined effects of the large distance between the SVC inlet and proximal row in this smaller venous geometry and the more favorable pressure drop between the SVC and tricuspid annulus at the higher SVC inlet flow rate.

**FIGURE 8:**
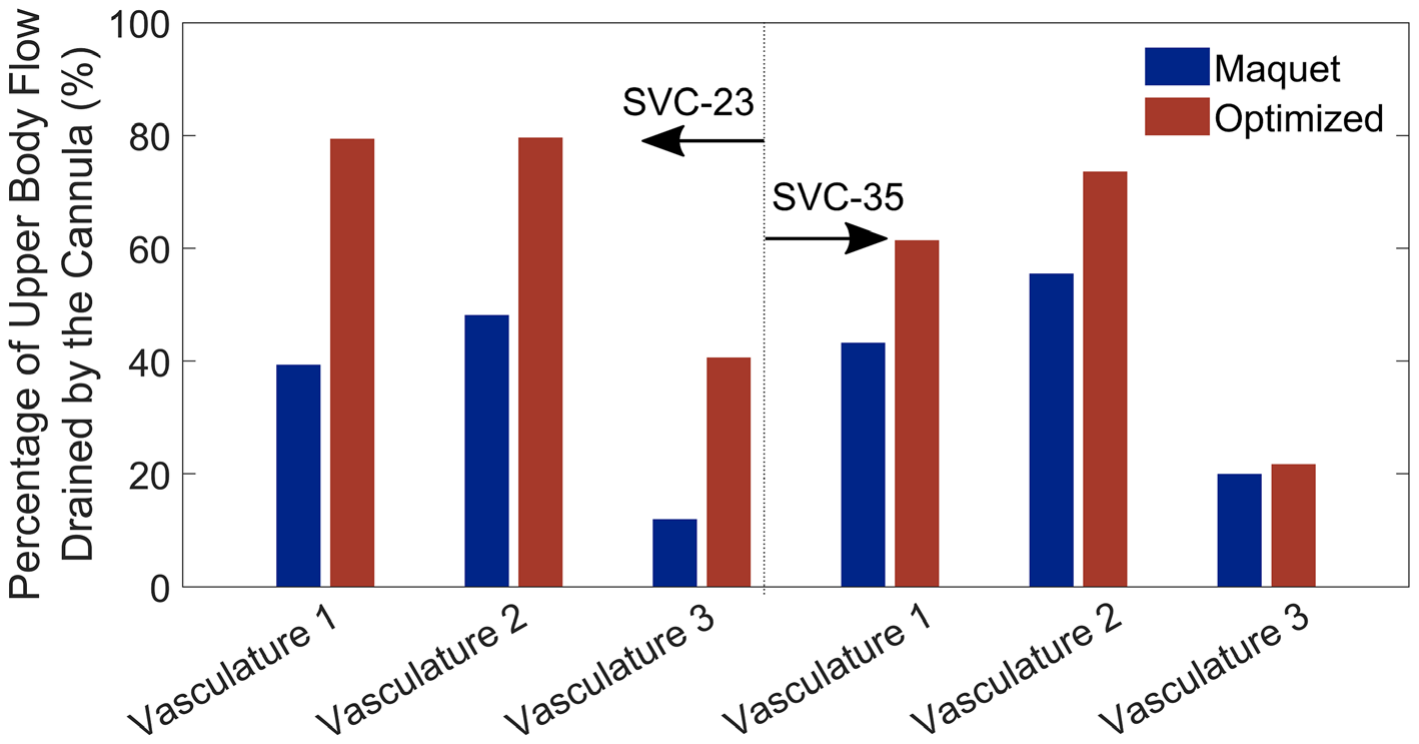
Drainage of upper body flow by the Maquet and optimized cannulas.

## 4 Discussion

This CFD study utilized an extensive optimization process, involving *n* = 540 DoE cannula designs, to generate a drainage cannula design that was optimized to improve flow dynamics. When tested in three different patient specific geometries with two different venous return distributions, the optimized cannula demonstrated lower pressure drops, increased cannula tip velocities, reduced blood stagnation volumes, lower hemolysis indices, faster rates of washout, and increased drainage from the upper body circulation during most of the simulated scenarios. Together, these improvements may reduce the risk of adverse events such as thrombosis and differential oxygenation in a clinical setting.

Drainage cannulas are associated with a high thrombosis risk during VA ECMO [3, 24]. Although the pathogenesis of the thrombi itself is multifactorial, abnormal flow dynamics can be a major contributor [3, 25]. The optimized cannula developed in this study often demonstrated lower blood stagnation volumes in the venous vasculature than the Maquet design. Increased flow through the tip and distal side holes ensured drainage of blood from the right atrial appendage, which was previously shown to be a high‐risk region for blood stagnation [6, 26]. This can be attributed to the reduction in the number of side hole rows and distal side hole diameters. This also reduces the risk of flow stasis at the distal end of the cannula, preventing the accumulation and deposition of activated platelets in these low‐velocity regions [27]. The tips of clinically available cannulas are particularly prone to thrombus formation, and clots are commonly observed there upon decannulation [1, 28]. Our results also revealed improved rates of washout in Vasculature 2, indicating a faster clearance of blood within the venous system when compared to the Maquet cannula.

The optimization process was also able to reduce the pressure drop associated with the optimized cannula. This improvement when compared to the Maquet cannula may reduce the overall circuit resistance to a point where pump speeds can be reduced without compromising on flow. A previous study demonstrated a similar reduction in pressure drop magnitudes when changing from a longer 55 cm cannula to a shorter 38 cm cannula [29]. Therefore, while cannula‐related hemolysis indices demonstrated minimal differences, a reduction in total circuit resistance may reduce the risk of pump‐associated blood trauma. However, further in vitro testing is required to confirm these benefits. The improved pressure drop in the optimized cannula can be attributed to the five large proximal row side holes, the reduced number of side hole rows, and smaller side holes in the distal row, which enabled increased flow from the tip end. Goto et al. [30] demonstrated that increased side hole numbers further limited the drainage flow rate, increased WSS, and reduced the cannula's tip velocity. In fact, a cannula with no side holes demonstrated the lowest WSS and higher drainage flow rate in their study [30]. Flow separation at each side hole due to the sharp change in flow direction introduces energy loss, which worsens potential flows and is exacerbated in cannulas with more side holes [13, 30]. This is also prothrombotic due to high shear rates between the separation bubble and the bulk flow, with further risk of activated platelet deposition within the separation bubble itself. Consequently, inclination of the side hole may enable a more favorable flow pathway. For instance, Park [13] demonstrated increased flow and Vatani et al. [15] demonstrated reduced WSS in cannulas with angled side holes, when compared to a perpendicularly inclined side hole. Interestingly, our optimization process chose a side hole angle of 31.5° as the preferred value. This is similar to the angle (30°) that had favorable outcomes and was ultimately chosen in the improved design by Vatani et al. [15]. Implementation of angled side holes would require alteration to the current dip molding process of cannula manufacturing such as inclination of the punch out mechanism that cuts the side holes.

The position of the proximal rows of side holes is crucial as they extract a majority of venous return, thereby impacting surrounding flow dynamics and subsequent complication risks. Clinically available multi‐stage cannulas often consist of five or more rows of side holes, which drain most of the flow via the proximal rows that are located near the renal veins within the IVC. Reduced upper body drainage then predisposes an increased risk of differential oxygenation and hypoxemia in patients with respiratory failure. Cai et al. [31] attempted to circumvent this issue by advancing the drainage cannula into the upper RA or SVC and demonstrated increased upper body oxygen saturation as a result. Our previous numerical studies demonstrated that increased SVC drainage is accompanied by impaired hepatic vein drainage [7, 18]. But no such hepatic congestion was observed during in vivo animal and human studies [31, 32]. Regardless, our optimized cannula increases blood flow via the tip and distal side holes, thereby ensuring greater upper body drainage when compared to the Maquet cannula at the same tip position. Although improvements to drainage cannula design can be beneficial, differential oxygenation is primarily an issue associated with arterial cannula flow. Studies have demonstrated that increased arterial cannula flow rates and advancing the arterial cannula tip can push the mixing zone proximally, thereby improving upper body oxygenation [33]. Another strategy to reduce differential oxygenation involves conversion to a V‐AV ECMO configuration by introducing an additional return cannula through jugular cannulation [34]. However, this is a tedious change to implement and may introduce additional bleeding and infection risks from the new cannulation site.

Overall, there were some limitations associated with this study. For instance, the CFD setup modeled the venous walls as rigid structures. Therefore, it is difficult to ascertain any benefits or drawbacks of the optimized cannula towards reducing complications such as vessel collapse. Future in vitro experiments are required to test these effects. RA movements were also neglected as a result, but CFD studies with rigid RA walls have closely aligned results to those observed in vivo [35]. Modeling such complex wall movements is computationally intensive and may even introduce non‐physiological vessel behavior. Nonetheless, the exclusion of such dynamic movements and pulsatility may impact parameters such as washout and blood residence time due to more efficient expulsion of blood from the right atrium. Another limitation of the study was the evaluation of the optimized cannula at one drainage flow rate. Additionally, our study demonstrated superior flow dynamics of the Optimized design only when compared to the Maquet model. Future work is still required to determine the superiority of the developed design over other clinically available designs from other manufacturers. Consequently, its performance at a range of other flow rates, such as during weaning, is yet to be determined. Our study was also unable to incorporate all branches of the venous tree. The incoming flow from excluded veins such as the coronary sinus may impact flow within the vasculatures and therefore affect cannula performance. Moreover, while our study investigated two different venous inflow distributions, we acknowledge that many other distributions exist that may alter flow within the vasculature. At last, the hemocompatibility improvements of the optimized cannula have not yet been verified by ex vivo or in vivo research and will be the aim of future work.

## 5 Conclusion

This study aimed to create a new multi‐stage drainage cannula design via an extensive optimization process, before comparing the developed design against a clinically available Maquet model in three patient specific geometries. The optimized design exhibited an improved pressure drop, reduced thrombosis risk, and increased upper body drainage when compared to the Maquet cannula. This was also evident in a scenario that modeled a higher contribution of upper body venous return. When translated to a clinical setting, the use of the optimized drainage cannula may reduce the incidence of cannula related adverse events. Future work will test the developed design in ex vivo hemocompatibility studies and in an in vitro cardiovascular simulator.

## Author Contributions

A.W. writing – original draft, methodology, investigation, formal analysis, data curation, conceptualization. M.K. writing – review and editing, investigation, formal analysis. A.B. conceptualization, writing – review and editing, supervision. S.D.G. writing – review and editing, supervision, resources, funding acquisition, formal analysis, conceptualization.

## Funding

Avishka Wickramarachchi would like to acknowledge the support of the Prince Charles Hospital Foundation (RF2025‐ECR‐01). Shaun D. Gregory would like to acknowledge the support of the National Health and Medical Research Council, Australia (Investigator Grant 2016995) and National Heart Foundation, Australia (Fellowship number 106675). Aidan Burrell would like to acknowledge the support of the National Health and Medical Research Council (Investigator Grant 2010110). Mehrdad Khamooshi was supported by the Prince Charles Hospital Foundation (RF2024‐O‐02).

## Ethics Statement

Anonymized patient imaging was used in this article in accordance with ethics approval from The Alfred Ethics Committee (Project 70/19).

## Conflicts of Interest

The authors declare no conflicts of interest.

## Supporting information


**Figure S1:** Surface and volume mesh used for patient Vasculature 1.
**Figure S2:** Parameters used for the design optimization stage.
**Table S1:** Mesh independence results.
**Table S2:** Parameterization settings.

## Data Availability

The data that support the findings of this study are available from the corresponding author upon reasonable request.
