# Supplementary material for: An Optimized Multi‐Stage Drainage Cannula Design for Venoarterial Extracorporeal Membrane Oxygenation
Source: Artif Organs. 2026 Jul 19;50(10):1372–83. doi: 10.1111/aor.70203 (PMC13622927; doi:10.1111/aor.70203)
Supplement: Supplementary file 1 [file AOR-50-1372-s001.docx]

# Supplementary Material A: Mesh Independence Study for the Ideal Geometry Setup

ANSYS Fluent (Canonsburg, PA, USA) was used to discretize the idealized geometry into polyhedral elements. A local curvature sizing between 0.15 and 0.35 mm was applied to the cannula walls. Five and ten inflation layers with a first layer height of 0.04 mm and 0.01 mm were applied to the venous and cannula walls, respectively. The maximum cell length for the volume mesh was set at 0.45 mm. A mesh convergence study deemed a final mesh size of 2.04 million cells to be adequate as it had less than 5% error for the measured parameters outlined in Table S2.

Table S1: Mesh independence results

| **Parameter** | **2.0 Million Elements** | **6.3 Million Elements** | **Percentage Difference (%)** |
| --- | --- | --- | --- |
| Pressure drop (mmHg) | 75.19 | 75.17 | 0.69 |
| Cannula tip velocity (mm/s) | 4.48 | 4.27 | 0.59 |
| Average wall shear stress (Pa) | 19.66 | 19.88 | 1.08 |
| Blood stagnation volume (ml) | 23.38 | 24.41 | 4.20 |

# Supplementary Material B: Mesh Details Used for the Patient-Specific Geometries

The patient specific geometries (Vasculatures 1, 2, and 3) were meshed using ANSYS Fluent (Canonsburg, PA, USA). A minimum surface mesh element size of 0.1 mm was used on the walled surfaces. Five and ten inflation layers with a first layer height of 0.05 and 0.01 were applied to the venous and cannula walls, respectively. A minimum orthogonal quality of 0.15 was met with all generated meshes. The volume mesh was discretized with polyhedral elements, resulting in mesh sizes between 2.6 and 3.5 million elements between the different vasculature and cannula geometries (Figure S1).

Figure S1: Surface and volume mesh used for patient vasculature 1.

# Supplementary Material C: Parameterization Settings

The cannula geometry was parameterized as described in Table S1 and Figure S1 to be used as input parameters for this study. To reduce the number of parameters, the distance between the tip and the first and proximal rows was left constrained at 40 mm (L1) and 200 mm (L2), respectively. Similarly, any given side hole angle was applied to all side holes, instead of a row-specific angle. The generation of the third and fourth row of side holes from the tip was optional since previous research has shown that with less rows may produce superior flow dynamics (increased flow and reduced thrombosis risk) [1].

Table S2: Parameterization settings

| **Parameterization** | **Allowable values** |
| --- | --- |
| Instance of side hole row (Creation instance) | 1 or 0. If the value is set to 1, the row is generated; if the value is set to 0, the row is not generated; only applied to the third and fourth row from the tip. |
| Angle of side hole profile, relative to the cannula’s longitudinal axis | 0 to 70°, applied globally to all side holes in all rows. Maximum of 70° of chosen to prevent lower quality meshes at higher angles, and manufacturing difficulties in producing such an acute angle. |
| Side hole diameter | 1 to 3 mm, specific and different for each row and applied to all side holes in a given row |
| Number of side holes per row | 2 to 5, specific and different for each row and applied in a circular pattern with the longitudinal cannula axis as the center. Maximum of 5 was chosen to prevent overlap of side holes when 3 mm diameter dimensions were applied. |
| Distance between each row, relative to the side hole row closer to the tip (except for the fourth row from the tip, as this was relative to the proximal row) | 10 to 50 mm, specific and different for each row, but not applied to the first and proximal rows. Maximum distance of 50 mm was chosen to prevent accidental overlap of side holes from other rows. |

Figure S2: Parameters used for the design optimization stage

[1] A. Wickramarachchi, S. D. Gregory, A. J. Burrell, and M. Khamooshi, “Flow characterization of Maquet and Bio-Medicus multi-stage drainage cannulae during venoarterial extracorporeal membrane oxygenation,” Computers in Biology and Medicine, vol. 171, pp. 108135, 2024.
